# Exploration of a possible relationship between examiner stringency and personality factors in clinical assessments: a pilot study

**DOI:** 10.1186/s12909-014-0280-3

**Published:** 2014-12-31

**Authors:** Yvonne Finn, Peter Cantillon, Gerard Flaherty

**Affiliations:** School of Medicine, National University of Ireland, Galway, Ireland; Postal address: Clinical Science Institute, National University of Ireland, Galway, Ireland; Discipline of General Practice, School of Medicine, National University of Ireland, Galway, Ireland

**Keywords:** Clinical assessments, Inter-examiner variability, Examiner stringency, Personality factors

## Abstract

**Background:**

The reliability of clinical examinations is known to vary considerably. Inter-examiner variability is a key source of this variability. Some examiners consistently give lower scores to some candidates compared to other examiners and vice versa – the ‘hawk- dove’ effect. Stable examiner characteristics, such as personality factors, may influence examiner stringency. We investigated whether examiner stringency is related to personality factors.

**Methods:**

We recruited 12 examiners to view and score a video-recorded five station OSCE of six Year 1 undergraduate medical students at our institution. In addition examiners completed a validated personality questionnaire. Examiners’ markings were tested for statistically significant differences using non-parametric one way analysis of variance. The relationship between examiners’ markings and examiner personality factors was investigated using Spearman correlation coefficient.

**Results:**

At each station there was a statistically significant difference between examiners markings, confirming the presence of inter-examiner variability. Correlation analysis showed no association between stringency and any of the five major personality factors. When we omitted an outlier examiner we found a statistically significant negative correlation between examiner stringency and openness to experience with a correlation coefficients (rho) of – 0.66 (p = 0.03). Conversely there was a moderate positive correlation between examiner stringency and neuroticism with a correlation coefficient (rho) of 0.73 (p = 0.01).

**Conclusions:**

In this study we did not find any relationship between examiner stringency and examiner personality factors. However, following the elimination of an outlier examiner from the analysis, we found a significant relationship between examiner stringency and two of the big five personality factors (neuroticism and openness to experience). The significance of this outlier is not known. As this was a small pilot study we recommend further studies in this field to investigate if there is a relationship between examiner stringency in clinical assessments and personality factors.

## Background

Reliability in assessment is “the reproducibility of assessment data or scores, over time or occasions” [1]. Traditional clinical assessments, such as the long case and the short case, have poor reliability and are unsuitable unless they are extended or combined with other assessments [2]. In response to this Harden et al. developed the objective structured clinical examination (OSCE) in the late 1970’s [2-4]. Since its introduction the OSCE has been widely accepted and used as an ‘objective’ method of assessing clinical skills. More recently, however, analyses carried out on many OSCEs have yielded unacceptably high levels of variability, with reliability coefficients ranging from 0.41 to 0.88 [5,6].

A key source of variability in clinical examinations is examiner variability. This refers to the fact that 2 examiners observing the same candidate’s performance at the same case may award different scores. This can be due to one examiner’s tendency to consistently give lower or higher scores compared with another examiner – termed examiner stringency or leniency and widely known as the hawk-dove effect. The second source of examiner variability is more random and includes examiner biases and the halo effect: these sources can be grouped under the term “rater error”, which Harasym defines as “individual idiosyncrasies associated with the random interaction effects of the rater, the testing situation, and the ratee [7]. More recently research into examiner cognition has identified variations in the cognitive processes of examiners, which may not be as random as previously thought, which contribute to inter-examiner variability; these processes include Differential Salience, Criterion Uncertainty and Information Integration [8].

Although the hawk-dove effect was described by Osler as far back as 1913 its impact on the reliability of clinical examinations has only been examined in recent years [9,10]. In 1974 Fleming et al. analyzed the MRCP (UK) examination and identified one examiner as a hawk, resulting in significantly lower pass rate in the group of candidates where this examiner was one of a pair of examiners compared with candidates in the group where this examiner did not examine (46.3% and 66.0% respectively) [11]. In 2003 Lawson analyzed 8 OSCEs administered in 2002 by the Canadian Chiropractic Examining board; this revealed that the variability due to examiner stringency was greater than the variability due to candidates [12]. Similarly Harasym et al. found a large difference in variance due to the hawk-dove effect (44.2%) in a communications skills scores in OSCEs [7]. Hill et al. examined the reliability of the mini-clinical evaluation exercise (mini-CEX), a workplace-based assessment, and found that 29% of the score variation was due to examiner stringency [13]. Mc Manus et al. analyzed the reliability of the Membership of the Royal College of Physicians (MRCP) UK clinical examinations between 2001 and 2004 and found that 12% of the variability was due to the hawk-dove effect [14].

The consequences of the effect of the hawk-dove effect on candidates who scored near the pass/fail mark in the Canadian Chiropractic board OSCE examinations was analyzed by Lawson. He found 6% of candidates in the pass/fail category had their scores changed from pass to fail or vice versa following adjustment for the hawk-dove effect [12]. In their study on the high-stakes MRCP(UK) examination McManus et al. found that, following adjustment for the hawk-dove effect, 2.65% of the candidates who failed based on the raw data, had their scores increased at or above the pass mark [14]. These studies demonstrate that examiner stringency can have significant consequences on the results of high stakes assessments for some candidates.

McManus demonstrated that examiner stringency over time was stable and hypothesized that it could be related to personality factors [14,15]. Personality traits are “stable aspects of personality that influence behavior in a wide range of settings” [16]. People have degrees of a trait i.e. they occupy a point along a continuous dimension, whereby a personality trait describes, “relative to other people, the frequency or intensity of a person’s thoughts, feelings or behaviors” [17]. Examiner stringency describes an examiner’s degree of stringency when compared with other examiners, and this also occupies a point along a continuum. This means that both personality traits and examiner stringency are relative i.e. they are considered in comparison with other persons and examiners respectively. These similarities between examiner stringency and personality traits, coupled with the gap in the literature on this topic, led us to conduct this pilot study to investigate if there is evidence to suggest a correlation between examiner stringency and personality factors. Our research question is “Does examiner stringency correlate with examiner personality factors?”

## Methods

Students in the foundation year of our undergraduate medical school complete an introductory course in professionalism and clinical skills in semester one. A five station OSCE is employed to assess clinical skills on completion of the module. This OSCE examines a narrow range of skills taught in this module, comprising communication skills and procedural skills such as vital signs and basic life support. For the purpose of this study an experimental five station OSCE which was congruent with the content and learning objectives of this module, was created. The skills tested were blood pressure measurement, communication skills (explaining a prescription), basic life support, communication skills (giving patient information about a planned endoscopy procedure), and hand-washing. The blood pressure measurement station and the two communication skills stations involved simulated patients. A mannequin was employed at the basic life support station. At the start of the second semester all 60 students in the Foundation Year were invited per email to participate in the study. Eight students responded and six of these were randomly selected to participate in the study. Candidate participants were provided with a participant information sheet and signed a consent form. The OSCE was recorded using digital cameras and a DVD was produced of the complete OSCE recording. Recruitment of examiners likewise involved inviting all examiners (38) from the pool of examiners who were eligible to examine medical students on the constructs included in this OSCE. Invites were sent from the faculty office per email, with responses directed to the study researchers. Thirty eight examiners responded to the invitation and twelve of these were randomly selected to participate in the study. Each examiner was provided with a participant information leaflet and signed a consent form. No examiner training was offered to recruited examiners prior to their participation in the study.

Examiners viewed the recorded OSCE at 1 of 4 prearranged viewing dates. The examiner’s pack consisted of:

1. An information sheet, indicating the instructions given to the candidates and instructions for the examiner on the skill and appropriate level of competency, was provided for each station. Marking sheets, consisting of a checklist and a global rating scale (GRS), were provided.
2. A sheet requesting demographic information on each examiner.
3. A personality questionnaire (the IPIP-NEO short form) consisting of 120 questions.

The IPIP-NEO short form is a validated personality questionnaire based on the five Factor model of personality as described by Costa and McCrae (the big five) [18]. Personality test results are presented as percentile estimates. For example, a person with a score of 60 in extraversion means he or she has a level of extraversion that is higher than 60% of persons of the same sex and age.

Examiners were given instructions prior to viewing the OSCE and, it was emphasized that there be no communication between examiners during the viewing and rating of candidates. This was a fully crossed design, whereby all examiners rated all student performances at each of the five stations. All data collected were coded for candidates and examiners; this was achieved by coding of examiner packs in advance of the viewing dates.

Ethics committee approval was obtained from Galway University Hospitals Ethics Committee, reference C.A. 508.

Data were analysed using SPSS 18.0. Summary statistics of examiner scores at each station, using median and interquartile ranges, were calculated and one-way repeated measures analysis of variance was conducted (Friedman test) at each station. Correlations between examiner total median scores and percentile estimates of the 5 major personality domains were calculated using Spearman’s correlation coefficient for non-parametric variables. P values < 0.05 were accepted as significant.

## Results and discussion

Six of the examiners were experienced clinical examiners, with 5 or more years examining at undergraduate and/or postgraduate assessments. The remaining six were less experienced and were all lecturers in the School of Medicine. There were nine male and three female examiners. All examiners were of Irish nationality. The six candidates recruited from foundation year were all female; five of them were Caucasian and the remaining candidate was non-Caucasian.

Preliminary analyses confirmed that the data were not normally distributed and, therefore, non-parametric methods were employed in the statistical analysis. The total possible score at each station was 40 marks. Table 1 shows median scores and interquartile ranges awarded by examiners at each station. This table demonstrates that there was considerable variability in examiners’ scores in this fully crossed assessment, whereby all examiners marked the same (and all) candidate performances in the recorded OSCE. The one-way repeated measures analysis of variance for non-parametric data (Friedman test) indicated that there was a statistically significant difference between examiners’ markings at each of the five stations (Table 2).

**Table 1:** **Examiner marks awarded to candidates at each of the five stations**

**Examiner**	**Blood pressure measurement**	**Communication skills (explaining a prescription)**	**Basic life support**	**Communication skills (explaining a planned endoscopy procedure)**	**Hand-washing**
1	26.5 (21.7-29.0)	23.0 (14.0-32.5)	30.5 (28.4-35.8)	32.0 (29.0-33.0)	35.7 (34.3-38.6)
2	27.5 (20.2-29.5)	16.5 (10.5-27.5)	35.2 (33.4-37.6)	29.0 (23.5-32.0)	34.3 (28.1-34.5)
3	23.5 (10.7-26.0)	28.0 (20.5-34.0)	34.2 (27.8-38.4)	39.0 (34.0-40.0)	36.6 (33.5-38.5)
4	25.0 (17.0-29.5)	18.0 (5.0-23.5)	37.9 (37.6-39.1)	34.0 (23.0-34.0)	33.3 (31.2-36.7)
5	24.5 (20.5-27.2)	23.0 (11.0-32.0)	27.9 (22.9-30.2)	27.0 (22.0-31.5)	31.4 (29.5-33.3)
6	22.5 (17.0-28.0)	25.0 (17.0-35.0)	32.1 (30.2-34.9)	30.0 (22.0-38.5)	38.1 (37.8-38.2)
7	29.5 (20.0-31.0)	24.0 (16.0-32.5)	33.1 (32.6-33.9)	30.0 (27.5-32.5)	34.7 (30.9-35.2)
8	27.0 (20.5-28.5)	21.0 (15.0-29.0)	30.0 (27.4-33.0)	27.0 (20.5-32.0)	27.6 (26.2-30.5)
9	20.5 (14.0-24.2)	25.0 (20.5-33.0)	31.0 (26.3-32.6)	31.0 (25.5-35.0)	34.3 (29.5-36.4)
10	10.0 (6.7-18.2)	26.0 (9.0-30.5)	27.4 (23.7-28.1)	28.0 (18.0-35.0)	22.4 (16.9-28.6)
11	15.0 (11.5-22.2)	17.0 (12.5-26.0)	30.5 (30.2-36.3)	32.0 (24.0-34.0)	36.2 (33.5-37.1)
12	13 (10.7-15.2)	15.0 (11.5-20.5)	15.8 (14.7-18.1)	16.0 (14.0-16.5)	16.1 (13.8-17.5)

Values are medians with interquartile ranges.

**Table 2:** **One-way repeated measures analysis of variance (Friedman test) of examiners markings at each station**

**Station**	**Chi-square**	**df**	**p value**
Blood Pressure Measurement	45.5	11	<0.0005
Communication skills (explaining a prescription)	34.7	11	<0.0005
Basic life support	50.4	11	<0.0005
Communication skills (explaining a planned endoscopy procedure)	34.4	11	<0.0005
Hand-washing	48.6	11	<0.0005

*Abbreviation*: *df* Degrees of freedom.

**Figure 1:**
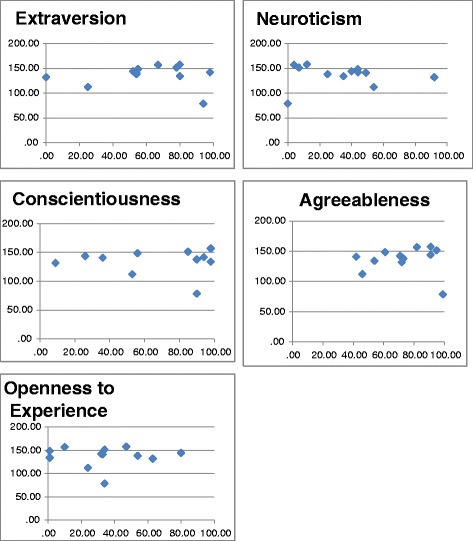
**Scatter plots of examiner scores and examiner percentiles of personality factors.** Personality factor percentiles are on the X-axis and examiner median scores on the Y-axis.

Personality testing revealed that our examiners had high median percentiles on extraversion (61), agreeableness (73) and conscientiousness (88). Median percentiles were lower on neuroticism (37) and openness to experience (34). Scatter plots of examiner median scores against each of the big five personality factors are shown in Figure 1. A significant relationship between examiner scorings and examiner personality factors was not found (Table 3). We noted an outlier observation, in each of the five scatter plots (Figure 1). This corresponded to the same examiner in the five scatter plots. As our sample size was small (12 examiners) this outlier had the potential to have a large impact on calculation of correlation coefficients between examiner stringency and personality factors. For this reason we performed a *posthoc* follow up analysis on the data omitting the data of the observed outlier. Analysis excluding the outlier, revealed a moderate negative correlation between examiner stringency and openness to experience with a Spearman’s correlation coefficient of – 0.66 (p = 0.03). There was a trend towards a moderate negative correlation between examiner stringency and extraversion with a rho value of – 0.53, although this did not reach statistical significance (p = 0.09). Neuroticism, on the other hand, had a moderate positive correlation with examiner stringency, with a correlation coefficient of 0.73 (p = 0.01).

**Table 3:** **Relationship between examiner scores and examiner personality factors**

**Domain**	**Data from all 12 Examiners**		**Data from 11 examiners – excluding outlier**	
	**Spearman’s correlation coefficient rho**	***p*** **value**	**Spearman’s correlation coefficient rho**	***p*** **value**
Extraversion	– 0.33	0.49	– 0.53	0.09
Agreeableness	0.04	0.90	0.01	0.97
Conscientiousness	– 0.34	0.28	– 0.45	0.16
Neuroticism	0.33	0.29	0.73	0.01*
Openness to Experience	– 0.27	0.39	– 0.66	0.03*

*Denotes a statistically significant correlation.

The significance of the identified outlier is unclear in this small study. It may represent a respondent who was “deliberately malingering, giving wrong answers, or simply did not understand the question on the questionnaire. On the other hand, it may be that the outlier is real and simply different” [19]. If our outlier is the former then the correlations excluding this participant may represent real associations between examiner stringency and the personality factors neuroticism and openness to experience. If our outlier is the latter, then, this participant may represent a group of examiners whose judgements in clinical examinations are not influenced by personality factors.

There is little research published on possible associations with examiner stringency, including personality factors. In their study on the MRCP(UK) clinical examinations McManus et al. found a positive association between examiner stringency and (a) the number of candidates examined, and (b) examiners from an ethnic minority [13]. No significant difference in stringency was found between male and female examiners or age of examiners. Harasym et al. (2008) found no difference in examiner stringency/leniency by level of training and by gender [12]. This pilot study opens a gap in the research and could inform design of larger follow up studies in this field.

A number of suggestions, including examiner training, have been made to improve inter-rater variability. Some studies in this field have failed to demonstrate improvements in inter-examiner score variability [20,21]. Arranging of examiner pairings in high-stakes examinations has also been proposed: McManus has suggested “pairing of high and low stringency examiners, so that raw marks can then be used in the determination of pass and fail” [13]. The marks of both examiners could then be averaged and this score awarded to the candidate. Another suggestion by McManus is adjustment of raw data scores to reduce the effect of examiner variability, using statistical methods such as generalisability theory or item response theory.

## Conclusions

Ours was a relatively small single site study that overall shows no significant relationships between examiner stringency and personality factors. Following exclusion of an outlying examiner our data suggest that the personality factors neuroticism and openness to experience may be associated with examiner stringency. If these relationships are confirmed in further studies they may add to our understanding of hawks and doves. In addition, if there is a relationship between personality factors and examiner stringency we suggest that personality testing may have a role to play in predicting the scoring behavior of examiners. This could be a factor to help guide conveners of clinical assessments in assigning new examiners, where there is little or no information on their level of stringency, with other examiners in examiner pairings. In the case where a relationship between personality and examiner stringency is supported further research would be required to determine if the relationship is an association only or if, indeed, personality factors influence examiner stringency.

## References

[1] Downing SM (2004). Reliability: on the reproducibility of assessment data. Med Educ.

[2] Wilkinson TJ, Campbell PJ, Judd SJ (2008). Reliability of the long case. Med Educ.

[3] Harden RM, Stevenson M, Downie WW, Wilson GM (1975). Assessment of clinical competence using objective structured examination. Br Med J.

[4] Ponnamperuma GG, Karunathilake IM, McAleer S, Davis MH (2009). The long case and its modifications: a literature review. Med Educ.

[5] Walters K, Osborn D, Raven P (2005). The development, validity and reliability of a multimodality objective structured clinical examination in psychiatry. Med Educ.

[6] Turner JL, Dankoski ME (2008). Objective structured clinical exams: a critical review. Fam Med.

[7] Harasym PH, Woloschuk W, Cunning L (2008). Undesired variance due to examiner stringency/leniency effect in communication skill scores assessed in OSCEs. Adv Health Sci Educ Theory Pract.

[8] Yeates P, O’Neill P, Mann K, Eva K (2013). Seeing the same thing differently: mechanisms that contribute to assessor differences in directly-observed performance assessments. Adv Health Sci Educ Theory Pract.

[9] Osler W (1913). Examinations, examiners and examinees. Lancet.

[10] Rushforth HE (2007). Objective structured clinical examination (OSCE): review of literature and implications for nursing education. Nurse Educ Today.

[11] Fleming PR, Manderson WG, Matthews MB, Sanderson PH, Stokes JF (1974). Evolution of an examination: M.R.C.P. (U.K.). Br Med J.

[12] Lawson D (2003). Use of the multifaceted rasch model to adjust for the error variance due to the examiner stringency-leniency effects in OSCEs.

[13] Hill F, Kendall K, Galbraith K, Crossley J (2009). Implementing the undergraduate mini-CEX: a tailored approach at Southampton University. Med Educ.

[14] McManus IC, Thompson M, Mollon J (2006). Assessment of examiner leniency and stringency ('hawk-dove effect') in the MRCP(UK) clinical examination (PACES) using multi-facet Rasch modelling. BMC Med Educ.

[15] McManus IC, Elder AT, Dacre J (2013). Investigating possible ethnicity and sex bias in clinical examiners: an analysis of data from the MRCP(UK) PACES and nPACES examinations. BMC Med Educ.

[16] Carver CS, Sheier MF: **The trait Perspective:** In *Perspectives on Personality.* 7th edition, International Edition. Boston: Pearson; 2012: 51–81.

[17] Costa PT, McCrae RR (1985). The NEO Personality Inventory manual.

[18] Johnson JA **Short Form for the IPIP-NEO (International Personality Item Pool Representation of the NEO PI-R™).** http://www.personal.psu.edu/~j5j/IPIP/ipipneo120.htm.

[19] Stockburger D: **Introductory Statistics: Concepts, Models, and Applications.** 2013. [http://www.psychstat.missouristate.edu/IntroBook3/sbk.htm]

[20] Holmboe ES, Hawkins RE, Huot SJ (2004). Effects of training in direct observation of medical residents’ clinical competence. Ann Intern Med.

[21] Cook DA, Dupras DM, Beckman TJ, Thomas KG, Pankratz VS: **Effect of rater training on reliability and accuracy of mini-CEX scores: a randomized, controlled trial.** *J Gen Intern Med* 2009, **24**(1):74–9. 10.1007/s11606-008-0842-3 PMC2607488 19002533

